# *Selaginella* extracts extend lifespan and mitigate oxidative stress in *Caenorhabditis elegans*

**DOI:** 10.3389/fphar.2025.1658991

**Published:** 2025-11-25

**Authors:** Xueqing Chen, Bingjian Yan, Yingmei Wu, Wenjing Quan, Yu Liu, Yaning Huang, Yinjie Shao, Yuan Wang, Qi Zhou, Yifei Liu, Songlin Liu, Jin Wang, Pan Li, Zhaohua Shi, Peng Shu, Junbo Gou

**Affiliations:** 1 HBN Research Institute and Biological Laboratory, Shenzhen Hujia Technology Co., Ltd., Shenzhen, China; 2 Hubei Shizhen Laboratory, Hubei Key Laboratory of Resources and Chemistry of Chinese Medicine, College of Pharmacy, Hubei University of Chinese Medicine, Wuhan, China; 3 Western Herbs (Hubei) Biotechnology Co., Ltd., Wuhan, China; 4 College of Food Science, South China Agricultural University, Guangzhou, China

**Keywords:** amentoflavone, *Caenorhabditis elegans*, DAF-16/FOXO pathway, lifespan, selaginella tamariscina, transcriptome

## Abstract

**Introduction:**

*Selaginella* species hold a traditional place in medicine and cosmetics, but their potential to extend lifespan and the underlying bioactive compounds remain inadequately investigated. This study aims to systematically evaluate the anti-aging properties of diverse Selaginella extracts and to identify the key bioactive components and mechanisms involved.

**Methods:**

We collected 23 *Selaginella* samples from 13 different provinces across China to assess their geographical influence. Two representative methanol extracts, S4 (high in amentoflavone) and S16 (low in amentoflavone), were selected for in-depth evaluation using the *Caenorhabditis elegans* model. We employed lifespan assays, stress resistance tests, and comparative transcriptomics to analyze the effects on longevity, and pathway modulation.

**Results:**

Both S4 and S16 extracts significantly extended the lifespan of *C. elegans* under normal conditions and modulated conserved longevity pathways, including MAPK and FOXO signaling, with daf-16 and *egl-8* emerging as key hub genes. Amentoflavone was identified and validated as a critical bioactive component, which alone extended lifespan by 63.81% and enhanced stress resistance. Mechanistically, amentoflavone promoted the nuclear translocation of DAF-16 and up-regulated the expression of antioxidant genes (e.g., *sod-3*, *gst-3/4*, *hsp-16.48/12.6*), leading to a significant reduction in intracellular ROS levels.

**Discussion:**

Our findings demonstrate that *Selaginella* extract and its key component, amentoflavone, delay aging primarily by activating the DAF-16/FOXO transcription factor and bolstering the antioxidant defense system. This study not only highlights amentoflavone as a major contributor to the lifespan-extending effects of Selaginella but also underscores the potential of these natural compounds as promising agents for healthy aging.

## 1 Introduction

Advancements in living standards and medical technology have markedly increased human life expectancy, resulting in a global demographic transition towards an aging population (Piggott and Woodland, 2016). The World Health Organization estimates that by 2050, adults aged 65 and above will account for 16% of the global population (Padeiro et al., 2023). Aging is associated with a progressive deterioration of physiological functions and mobility, compromising the body’s capacity to maintain homeostasis and recover from stress (Guo et al., 2022). This decline elevates the risk of numerous age-related diseases, such as diabetes, cardiovascular diseases, and Parkinson’s disease (Guo et al., 2022). This functional decline significantly elevates the susceptibility to age-related pathologies, including diabetes, cardiovascular disorders, and neurodegenerative conditions such as Parkinson’s disease (Guo et al., 2022). The escalating prevalence of these diseases imposes substantial societal and economic burdens, underscoring the urgent need for effective strategies to delay aging and enhance healthspan (Jarzebski et al., 2021). Consequently, identifying interventions that mitigate aging and improve quality of life in the elderly has emerged as a paramount research objective worldwide.

In recent years, Chinese herbal medicine (CHM) has attracted growing interest for its potential lifespan extension benefits (Zhao et al., 2020). Numerous medicinal plants—such as *Psoralea corylifolia* (Wang et al., 2022), *Salsola collina* (Wang et al., 2024), and *Salvia haenkei* (Zumerle et al., 2024)—along with their bioactive constituents, including corylin, luteolin, quercetin, β-sitosterol, and salicylic acid, have demonstrated efficacy in extending lifespan and attenuating aging-related decline. Among these, the genus *Selaginella* has garnered particular attention due to its abundance of biflavonoids, especially amentoflavone and its derivatives, which exhibit broad applications in traditional medicine and cosmetics (Křížkovská et al., 2020; Kumar et al., 2021; Bailly, 2021). For instance, *S. tamariscina* is extensively utilized in Asian traditional medicine for treating hemorrhage, inflammation, immune dysregulation, cancer, oxidative stress, hyperglycemia, and hypercholesterolemia (Bailly, 2021). Additionally, *S. rossii* has been shown to confer protection against skin aging and UVB-induced wrinkling via its antioxidant activity (Lee et al., 2022). Despite these documented therapeutic effects, a systematic evaluation of the lifespan-extending potential of *Selaginella* species and the underlying molecular mechanisms remains lacking. This gap highlights the need for comprehensive investigations into the lifespan extension properties of *Selaginella*.

*Caenorhabditis elegans* has become an important biological model for studying the lifespan extension mechanisms of CHMs, including *Lonicera japonica*, *Lycium barbarum*, *Ganoderma lucidum*, *Astragalus membranaceus*, and *S. collina* (Wan et al., 2014; Zhang et al., 2020; Wang et al., 2021; Lin et al., 2023; Wang et al., 2024). Over the past few decades, numerous genes (e.g., *daf-2*, *daf-15*, *daf-16*, *daf-18*, *let-363*, *egl-8, lin-45*, etc.), genetic pathways (e.g., the insulin/insulinlike growth factor-1(IIS), mammalian target of rapamycin (mTOR), AMP-activated protein kinase (AMPK) signaling pathways, etc.), as well as many environmental factors (e.g., temperature, ultraviolet, oxidation exposures, etc.) contributing to the aging process, have been identified from studies using *C. elegans* (Cho and Park, 2024). In parallel, network pharmacology has emerged as a powerful tool in the study of traditional Chinese medicine (TCM) and functional foods, providing insights into the interactions between bioactive compounds and human biological systems (Zhang et al., 2023; Sha et al., 2024). It has been applied in various studies to investigate immune and aging regulatory mechanisms and therapeutic effects, such as in research on different rice varieties (Sha et al., 2024) and on *Rosmarinus officinalis* L. (Bisht et al., 2024). Combining *C. elegans* models with network pharmacology offers a promising approach to uncovering the mechanisms by which *Selaginella* and its bioactive components influence aging and longevity.

In this study, we collected 23 *Selaginella* samples from 13 provinces across China to assess their effects on aging and lifespan in *C. elegans*. Using comparative transcriptomics, we explored how geographical origin influences metabolic profiles and aging-related transcriptomic changes, while also identifying key bioactive compounds. Our results indicate that amentoflavone is a major contributor to the lifespan extension effects of *Selaginella*, likely through enhancing antioxidant defense via activation of the DAF-16/FOXO transcription factor. We further demonstrate that both *Selaginella* methanol extracts and purified amentoflavone extend lifespan and ameliorate aging in *C. elegans* by bolstering antioxidant mechanisms.

## 2 Materials and methods

### 2.1 Materials

*Selaginella* plant materials were collected from 13 provinces in China and cultivated at the understory resource nursery at Hubei University of Chinese Medicine. Details of the collection sites were listed in Supplementary Table S1 and Supplementary Figure S1, and morphological characteristics of select *Selaginella* specimens were shown in Supplementary Figure S2. Wild-type *C. elegans* (N2 strain), DAF-16 mutant worms (CF1038), DAF-16GFP (TJ356) and *Escherichia coli* OP50 were generously provided by Dr. Pan Li from South China Agricultural University.

For molecular experiments, the Total RNA Kit (RC112-01), and HiScript III first Strand cDNA Synthesis Kit (R312-01) were obtained from Vazyme Biotech Co., Ltd. (Nanjing, China). Astaxanthin (catalog number: SA8730), and amentoflavone (catalog number: 1,617-53-4) were sourced from Shanghai Yuanye Bio-Technology Co., Ltd. (Shanghai, China).

### 2.2 Preparation of *Selaginella* methanolic extracts (SMEs)

The aerial parts of *Selaginella* were harvested and dried at 60 °C until a constant weight was obtained. The dried plant material was then finely ground and passed through a 65-mesh sieve. For extraction, 100 mg of the powdered sample was soaked in 10 mL of methanol (HPLC Grade, ≥99.9%, FTSCI Hubei) for 40 min. This was followed by ultrasonic extraction using an SB-4200DT ultrasonic cleaner (SCIENTZ, China) at 55 °C for 30 min. The mixture was then filtered through a 0.45 µm membrane (XingYa, Shanghai) to obtain crude extracts, designated as MEs, at a final concentration of 10 mg mL^-1^. This ME solution was used as the stock solution and further diluted to 0.5, 1.0, and 2.0 mg mL^-1^ for subsequent experiments.

### 2.3 Preparation of amentoflavone monomer

Twenty milligrams of amentoflavone monomer were dissolved in methanol and diluted to a final concentration of 1 mg mL^-1^ to prepare a stock solution. This stock solution was then further diluted to concentrations of 25, 50, and 100 μg mL^-1^ for use in subsequent experiments. Astaxanthin was prepared separately by diluting in chloroform to a final concentration of 0.64 µM. All solutions were filtered using a 0.45 µm organic filter membrane, aliquoted into 500 µL portions, and stored at −80 °C until further use.

### 2.4 Culture and physiological indices of *C. elegans*

The *C. elegans* strains were cultured at 20 °C on solid Nematode Growth Medium (NGM) agar plates seeded with *Escherichia coli* OP50.

For the heat stress resistance assay, 60-69 worms were divided into each experimental group, with each group distributed across three NGM plates (20-23 worms per plate). The plates were supplemented with either 0.5, 1.0 and 2.0 mg mL^-1^ of *Selaginella* methanol extracts (SME) or 50 μg mL^-1^ of amentoflavone monomer. The worms were incubated at 37 °C, and the number of survivors was recorded at 1-hour intervals until all the worms perished.

Similarly, in the ultraviolet (UV) stress resistance assay, 60-69 worms were distributed into groups on three NGM plates (20-23 worms per plate), supplemented with either 0.5, 1.0 and 2.0 mg mL^-1^ of SME or 50 μg mL^-1^ of the monomer. The worms were then exposed to UV irradiation (8 W) for six additional days. Survival rates were recorded at 12-hour intervals until all the worms perished.

For lifespan analysis of wild-type *C. elegans* (N2 strain) or daf-16 mutant worms (CF1038), the assay were performed following previously described methods (Wang et al., 2024). Worms were synchronized and lysed, then cultured on NGM plates at 20 °C until reaching the L4 larval stage. The L4 larvae were exposed to SME or the monomer to evaluate lifespan. Worms treated with 100% methanol served as the negative control. To prevent progeny development, 5-fluorodeoxyuridine (10 μg mL^-1^) was administered from day 0 to day 5. The number of surviving worms was recorded daily, and the worms were transferred to fresh plates every 24 h. Survival curves were analyzed using GraphPad Prism 9 software.

### 2.5 Transcriptome sequencing analysis

Synchronized L4 *C. elegans* (N2 strain) were transferred to NGM plates containing the SMEs (0.5, 1.0, 2.0 mg mL^-1^) or monomer amentoflavone (50 μg mL^-1^). Worms were transferred to fresh NGM plates daily throughout the 5-day treatment period. Afterward, approximately 200 worms were harvested for RNA sequencing analysis.

Total RNA extraction followed the protocol of the R6834 Total RNA Kit I. Libraries were then prepared and sequenced using the DNBSEQ high-throughput sequencing platform (BENAGEN, China). The trimmed sequencing reads were aligned to the *C. elegans* reference genome (Yoshimura et al., 2019) using STAR software (version 2.7.9a). For transcript assembly, StringTie (version 2.1.4; default parameters) was employed, and the assembled transcripts were merged using StringTie’s “merge” function. The merged transcripts were compared with known genome annotations using gffcompare (version 0.12.1; parameters: R-C-K) to identify novel transcripts and genes, thereby complementing the existing annotations. Transcript abundance and gene quantification were calculated using RSEM software.

Transcript levels were quantified by calculating Fragments Per Kilobase of transcript per Million mapped reads (FPKM), with an FPKM value of 1 as the threshold for transcript expression. Differentially expressed transcripts (DGTs) were identified based on the criteria |logFC| > 1 and *p* < 0.05. Data visualization and functional enrichment analyses, including volcano plots, heatmaps, GO term enrichment, KEGG pathway analysis, and Gene Set Enrichment Analysis (GSEA), were performed using the *clusterProfiler* package.

To explore the interactions among the significant transcripts (*p* < 0.05), a PPI network was constructed. The PPI network was built using the Search Tool for the Retrieval of Interacting Genes (STRING) database (https://www.string-db.org/) and visualized using Cytoscape software.

### 2.6 High-performance liquid chromatography (HPLC) analysis

The HPLC analysis was carried out using an Agilent 1,260 Infinity II LC system (Agilent Technologies Inc.) equipped with an Agilent 5 TC-C18 (2) column (250 × 4.6 mm, 5 μm). The chromatographic conditions were as follows: solution A: 0.1% phosphoric acid water, solution B: 100% methanol (high performance liquid phase grade); Column temperature: 30 °C; Detection wavelength: 330 nm; Injection volume: 10 μL; Flow rate: 1 mL min^-1^; Gradient conditions: 0.0 min, 40% B; 14.0 min, 50% B; 19.0 min, 60% B; 24.0 min, 70% B; 29.0 min, 80% B; 49.0 min, 90% B; 51.0 min, 40% B; 53.0 min, 40% B.

### 2.7 Pharyngeal aspiration, body bending and fertility indices

Nematodes were maintained under the same culture conditions as described in the lifespan assay. The pharyngeal pumping rate was assessed following a previously reported method (Ding et al., 2024), with slight modifications. Briefly, on days 3 and 7 of cultivation, relaxation and contraction cycles of the pharyngeal pump were observed and counted over a 30-second interval under a microscope. In addition, body bending frequency was evaluated on days 3, 7, and 12 of culture, during which body bends were recorded within a 30-second observation window (Yu et al., 2021).

For the fertility assay, synchronized nematodes were individually transferred to NGM plates—either blank control or amentoflavone (50 μg mL^-1^) treatment groups—with three replicates per group. Of note, 5-FU was not included in the NGM plates during this experiment. Every 24 h, the nematodes were transferred to fresh corresponding NGM plates. The number of eggs was recorded once the offspring had developed to the L2 or L3 larval stage, and counting continued until the end of the egg-laying period.

### 2.8 Quantitative RT-PCR (RT-qPCR) analysis

RNA was extracted from three biological replicates, each consisting of approximately 200 worms. Total RNA was isolated using Trizol reagent, and cDNA was synthesized with SuperScript^®^ II Reverse Transcriptase. RT-qPCR was performed using TB Green^®^ Premix Ex Taq™ II (Tli RNaseH Plus) on a StepOnePlus™ Real-Time PCR System, in accordance with the manufacturer’s instructions. The housekeeping gene *pmp-3* (GenBank accession number NM_001269678.3) was used as an internal control. Primer sequences are provided in Supplementary Table S2.

### 2.9 Measurement of ROS levels in *C. elegans*

Intracellular ROS levels were detected according to the method described by Li et al. (2022). N2 wild-type worms were treated with methanol or amentoflavone for 5 days. Subsequently, the worms were collected into 1.5 mL centrifuge tubes using M9 buffer and washed three times. The nematodes were then incubated with 2,7-dichlorodihydrofluorescein diacetate (H_2_DCF-DA; 1 μM, Sigma) at 20 °C for 4 h. After incubation, the worms were washed with M9 buffer, anesthetized with 5 mM levamisole, transferred onto glass slides, and visualized under a fluorescence microscope (Olympus IX73, Tokyo, Japan) at an excitation wavelength of 485 nm and an emission wavelength of 535 nm. Fluorescence intensity was quantified using ImageJ software (NIH, Bethesda, MD). Each experiment was performed in triplicate with 30 worms per treatment group.

### 2.10 Analysis of DAF-16 nuclear localization

The subcellular localization of DAF-16 was examined as previously reported (Lin et al., 2019). The transgenic strain TJ356 was employed to monitor DAF-16GFP localization. After treatment, worms were washed with M9 buffer and anesthetized using 5 mM levamisole. Images were acquired with a fluorescence microscope and analyzed using ImageJ. DAF-16GFP localization patterns were classified into three categories: cytosolic, intermediate, and nuclear. Assays were conducted in triplicate, with 30 worms analyzed per treatment.

### 2.11 Statistical analysis

All data were analyzed using GraphPad Prism 9.0. The Log-rank (Mantel-Cox) test was employed for survival analysis, while comparisons between two groups were performed using a two-sided Student’s t-test. For multiple comparisons, two-way ANOVA with Bonferroni’s correction was applied. Results are presented as the mean ± standard deviation (SD). Statistical significance was set at *p* < 0.05, *p* < 0.01, *p* < 0.001, and *p* < 0.0001.

## 3 Results

### 3.1 Collection and analysis of *Selaginella* resources

**FIGURE 1:**
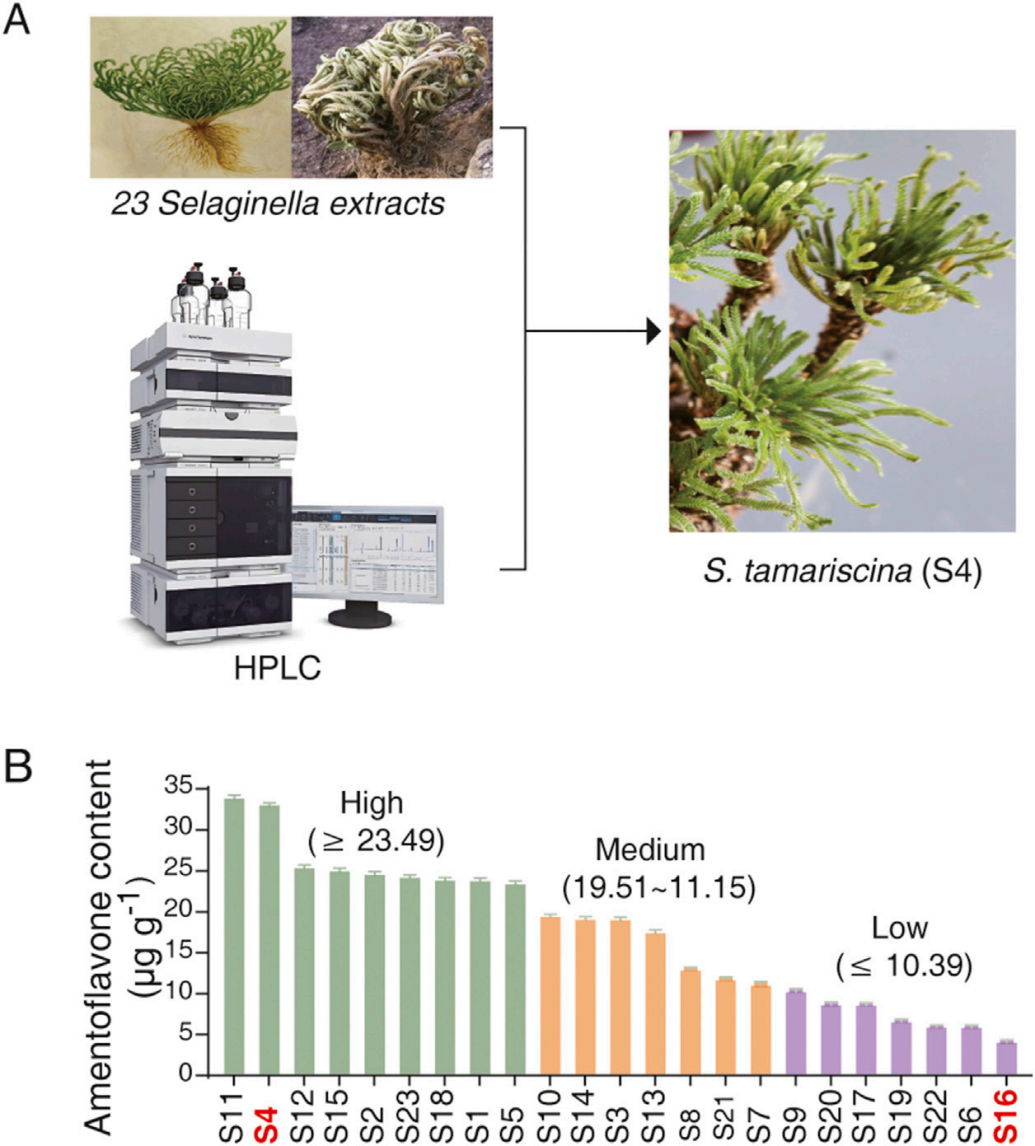
Collection and phytochemical profiling of *Selaginella* samples. **(A)** Geographic distribution of the 23 *Selaginella* samples collected from 14 provinces across China. **(B)** Amentoflavone content (µg g^-1^) in the 23 *Selaginella* samples as quantified by HPLC. Samples are categorized into three groups based on amentoflavone levels: high (≥23.92 μg g^-1^), medium (11.15–23.49 μg g^-1^), and low (≤10.39 μg g^-1^). S4 (high) and S16 (low) were selected for further study.

*Selaginella* has been widely utilized in traditional medicine and cosmetics due to its bioactive properties, which include promoting blood circulation, regulating menstruation, and enhancing skin whitening (Bailly, 2021; Lee et al., 2022). However, its potential effect on lifespan extension remains unexplored. To address this, we collected 23 *Selaginella* samples from 14 different provinces across China (Figure 1A; Supplementary Figures S1–S2; Supplementary Table S1), designated as S1 to S23. HPLC analysis revealed substantial variation in the concentration of amentoflavone—a key bioactive biflavonoid and an established chemical marker in *Selaginella* (Zhang et al., 2022)—across species and geographic origins (Figure 1B; Supplementary Table S3). Based on amentoflavone content, the samples were classified into three groups: high (≥23.92 μg g^-1^), medium (11.15–23.49 μg g^-1^), and low (≤10.39 μg g^-1^) (Figure 1B).

For subsequent lifespan analysis, two representative samples with contrasting amentoflavone levels were selected: S4, which had the second-highest content (33.07 μg g^-1^) from the high group, and S16, which had the lowest (4.24 μg g^-1^) from the low group. This comparative approach, based on divergent phytochemical profiles, allows for a more precise assessment of the potential role of *Selaginella* extract and its constituent amentoflavone in modulating lonevity.

### 3.2 *Selaginella* extract extends *C. elegans* lifespan

**FIGURE 2:**
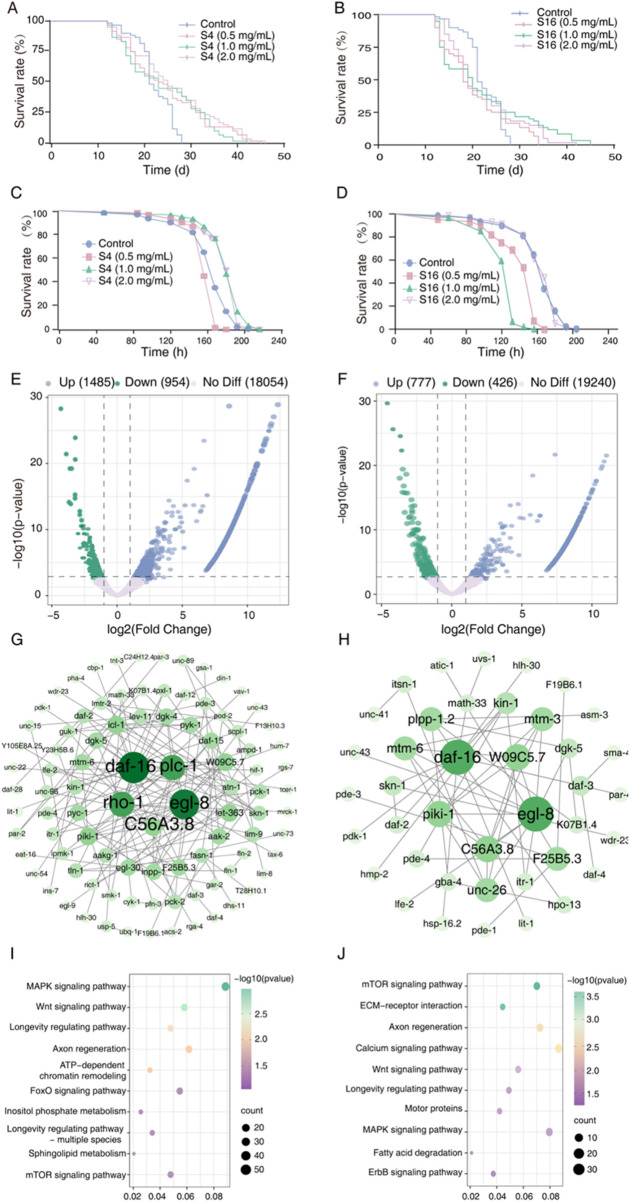
Effects of *Selaginellae herba* extracts on *C. elegans* lifespan and transcriptomic profiles. **(A,B)** Survival curves of nematodes treated with *Selaginellae herba* methanol extracts (SME) at indicated concentrations. BC: Blank control (methanol); S4L: 0.5 mg mL^-1^ S4 extract; S4M: 1 mg mL^-1^ S4; S4H: 2 mg mL^-1^ S4; S16L: 0.5 mg mL^-1^ S16; S16M: 1 mg mL^-1^ S16; S16H: 2 mg mL^-1^ S16. **(C,D)** Survival curves under UV stress following SME treatment (concentrations as in A, B). **(E,F)** Volcano plots of transcriptomic changes in S4 SEM-treated **(E)** and S16 SEM-treated **(F)** worms versus untreated controls. Genes with |log_2_FC| > 1 and p < 0.05 are highlighted (blue: upregulated; green: downregulated; pink: non-DEGs). **(G,H)** Protein-protein interaction networks of top differentially expressed transcripts (DETs) in control vs. S4 SEM **(G)** and S16 SEM **(H)** groups. **(I,J)** KEGG pathway enrichment analysis of significant DETs from S4 SEM **(I)** and S16 SEM **(J)** treatments. Statistical significance vs. methanol-treated control determined by one-way ANOVA with log-rank test (p < 0.05, p < 0.01, p < 0.001, *p <* 0.0001).

As lifespan is a primary biomarker of aging, we evaluated the lifespan extension potential of two distinct *Selaginella* extracts S4 and S16—in the *C. elegans* model using a concentration-gradient experiment (0.5, 1.0, and 2.0 mg mL^-1^). By day 21, lifespan curves for S4-treated worms exhibited significant rightward shifts compared to the methanol-treated control, corresponding to average lifespan extensions of 8.56%–17.19% (Figure 2A; Table 1). Similarly, by day 27, S16-treated groups showed rightward shifts relative to the control; however, these were less pronounced than those observed with S4, suggesting potential concentration-dependent toxicity in this specific geographical variant (Figure 2B; Table 1). These results demonstrate that *Selaginella* extracts significantly extend lifespan in wild-type *C. elegans*, with efficacy varying according to geographic origin and associated phytochemical composition.

**TABLE 1:** Lifespan extension in *C. elegans* treated with *Selaginella* extracts (S4 and S16) and amentoflavone under standard culture conditions.

Group	N	Mean ± SE	Maximum	Median	Rate(%)	*P-value*
Methanol	60	22.2 ± 0.50	28	21	-	-
S4L	60	24.76 ± 1.16	45	23.5	11.56	0.4922
S4M	60	24.1 ± 1.12	42	23	8.56	0.8522
S4H	60	26.01 ± 1.20	46	25	17.19	0.026
S16L	60	20.38 ± 0.89	34	18	−8.91	0.0001
S16M	60	22.1 ± 1.26	45	20	−0.45	0.0153
S16H	60	21.9 ± 0.96	42	19	−1.37	0.0253
Amentoflavone	60	36.36 ± 0.85	45	39	63.81	<0.0001

N, number of *C. elegans*.

### 3.3 *Selaginella* extract modulates stress resilience in *C. elegans*

To further assess the survival benefits of S4 and S16, we examined their effects on *C. elegans* under UV stress. S4 treatment resulted in significant rightward shifts in survival curves, increasing lifespan by 1.47%–10.81% under UV exposure (Figure 2C; Table 1). In contrast, low and medium concentrations of S16 caused leftward shifts in the survival curve. The high-concentration S16 group showed no difference from the control under UV stress (Figure 2D; Table 1). These findings indicate that S4 not only extends lifespan under normal conditions but also enhances resilience to UV stress, supporting its lifespan extension potential. Conversely, S16 failed to improve lifespan under UV stress.

### 3.4 Transcriptomic analysis reveals lifespan extension mechanisms of *Selaginella*

To investigate the lifespan extension mechanisms of *Selaginella* derivatives, we performed RNA-seq analysis on *C. elegans* treated with S4 or S16. S4 treatment induced 2,439 differentially expressed transcripts (DETs), significantly enriched in MAPK, FOXO, mTOR, and longevity-regulating pathways (Figure 2E; Supplementary Tables S3-S5). S16 treatment altered 1,203 DETs, predominantly enriched in MAPK, Wnt, ErbB, and longevity-regulating pathways (Figure 2F; Supplementary Tables S6-S8). Although S4 induced approximately twofold more DETs than S16, both treatments shared enrichment in MAPK and longevity-regulating pathways, suggesting a common lifespan extension mechanism. This result indicates that *Selaginella* derivatives extend lifespan through coordinated modulation of evolutionarily conserved longevity pathways and compound-specific metabolic interventions.

Protein interaction network analysis identified five hub genes per treatment, with *daf-16, egl-8,* and *C56A3.8* common to both groups (Figures 2G,H). Functional studies confirmed their critical roles: DAF-16 mediates insulin/IGF-1 signaling in lifespan regulation; *egl-8* (typically associated with nicotine dependence studies) encodes a protein that activates 1-phosphatidylinositol 4-kinase and participates in phosphatidylinositol phosphate biosynthesis. These findings suggest both samples converge on FOXO signaling activation (Figures 2I,J).

To identify potential bioactive components, we conducted network pharmacology and molecular docking analyses (Supplementary Figure S3-S6; Supplementary Table S9-S12). The results revealed ten key candidate compounds: andromedotoxin, asebotoxin, beta-caryophyllene, isocembrol, hinokiflavone, isocryptomerin, amentoflavone, selaginellin, hinokinin, and tremetone. Combined with HPLC analysis identifying the amentoflavone as primary active substance in *Selaginella*, we selected amentoflavone for further mechanistic study (Figure 1A).

### 3.5 Amentoflavone extend *C. elegans* lifespan

**FIGURE 3:**
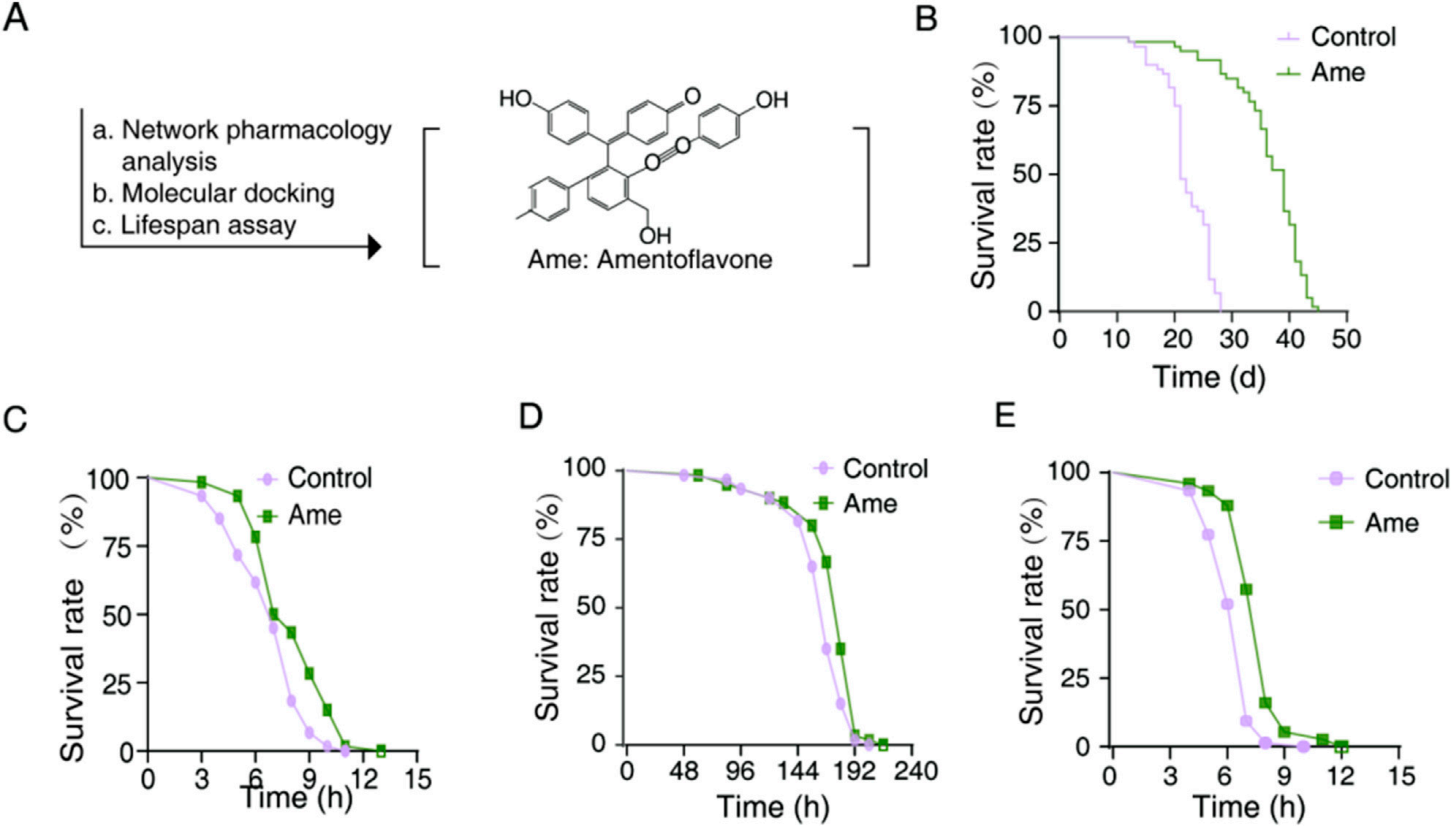
Amentoflavone extend lifespan and enhance the resilience to environmental stress of *C. elegans*. **(A)** Flow diagram of lifespan extension compositions from amentoflavone; **(B)** Survival curve of amentoflavone-treatment; **(C)** Survival of heat stress of amentoflavone-treatment **(D)** Survival of ultraviolet stress of amentoflavone-treatment **(E)** Survival of Oxidative Stress of amentoflavone-treatment. Ame, amentoflavone; The treated concentration of amentoflavone is 50 μg mL^-1^, compared to the mock-treated control by one-way ANOVA following log-rank test.

In pre-experimental tests, amentoflavone was evaluated at concentrations of 25, 50, and 100 μg mL^-1^. The 50 μg mL^-1^ concentration demonstrated the most pronounced lifespan extension without any adverse effects, such as alterations in feeding behavior, and was therefore chosen for further mechanistic investigation. Lifespan analysis of *C. elegans* treated with 50 μg mL^-1^ amentoflavone revealed a significant extension of 63.81% (Figure 3B; Table 1). This represents a 3.7-fold greater increase compared to the group treated with S4 SME, identifying amentoflavone as a key contributor to the lifespan-extending properties of *Selaginella*.

Further assessment under UV, thermal and oxidative stress conditions showed that amentoflavone induced rightward survival curve shifts (Wang et al., 2020; Hou et al., 2023). Lifespan enhancements ranged from 1.47% under UV stress, 18.29% under thermal stress and 19.75% under oxidative stress (Figures 3C–E; Tables 2-4). Notably, the improvement in both thermal and oxidative stress resistances were significantly greater than under UV stress, suggesting this amentoflavone may primarily extend lifespan by enhancing resistance to thermal and oxidative damage.

**TABLE 2:** Survival of *C. elegans* under ultraviolet (UV) stress following treatment with *Selaginella* extracts and amentoflavone.

Group	N	Mean ± SE	Median	Maximum	Rate (%)	*Pvalue*
Methanol	60	162.8 ± 3.63	168	204	-	-
Astaxanthin	60	177.4 ± 2.84	180	216	8.97	<0.001
S4L	60	157 ± 2.20	156	180	−3.56	ns
S4M	60	180.4 ± 3.24	180	216	10.81	ns
S4H	60	177.8 ± 3.22	186	216	9.21	ns
S16L	60	138.4 ± 3.74	156	168	−15.30	0.1251
S16M	60	123 ± 2.23	132	156	−24.72	0.0012
S16H	60	163.4 ± 3.27	168	204	−0.37	ns
Amentoflavone	60	172.2 ± 3.79	180	216	5.77	<0.001

N, number of *C. elegans*.

**TABLE 3:** Lifespan of *C. elegans* under heat stress after treatment with amentoflavone and astaxanthin.

Group	N	Mean ± SE	Median	Maximum	Rate (%)	*P value*
Methanol	60	6.83 ± 0.26	7	11	-	-
Astaxanthin	60	8.27 ± 0.25	8	13	20.98	<0.001
Amentoflavone	60	8.08 ± 0.26	7.5	13	18.29	<0.001

N, number of *C. elegans*.

**TABLE 4:** Lifespan of *C. elegans* under oxidative stress after treatment with amentoflavone and astaxanthin.

Group	N	Mean ± SE	Median	Maximum	Rate (%)	*Pvalue*
Methanol	60	6.34 ± 0.48	7	10	-	-
Astaxanthin	60	7.58 ± 0.65	8	12	19.54	<0.001
Amentoflavone	60	7.64 ± 0.65	7	12	20.38	<0.001

N, number of *C. elegans*.

Collectively, these findings demonstrate that amentoflavone significantly extend *C. elegans* lifespan and improve thermal and oxidative stress resistances, confirming its role as a key active compound in *Selaginella* for lifespan extension.

### 3.6 Amentoflavone increases lifespan of *C. elegans* by activating the DAF-16/FOXO transcription factor

**FIGURE 4:**
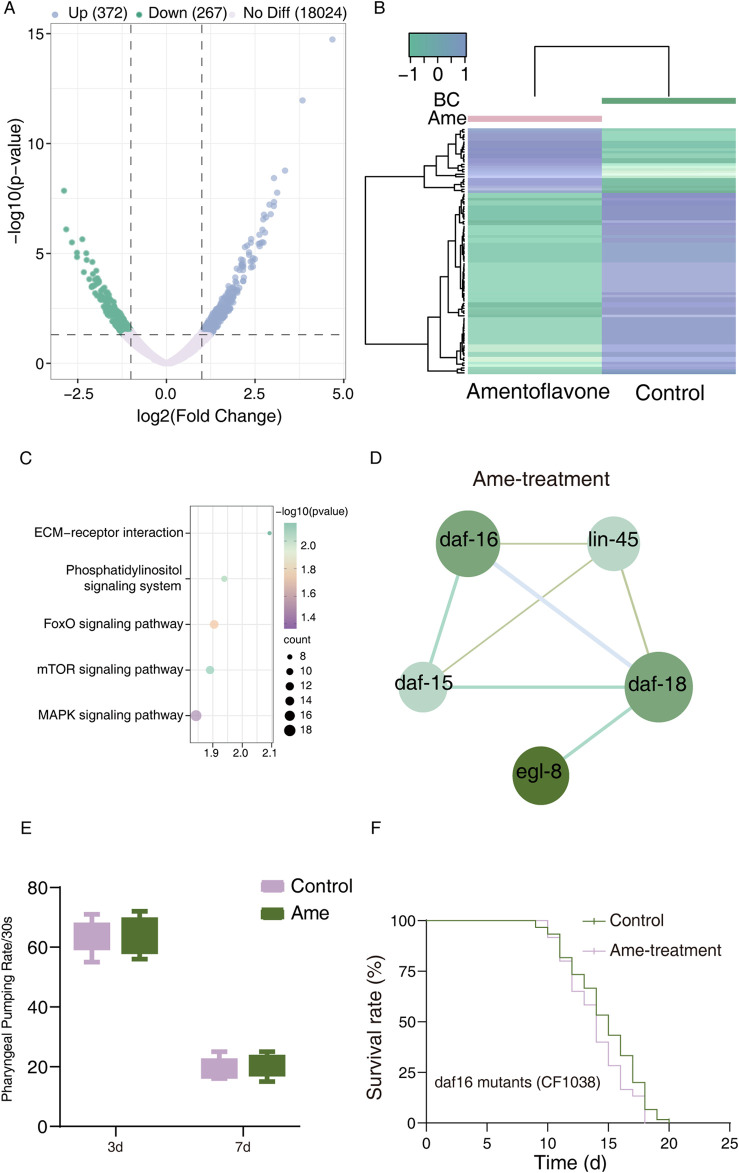
Transcriptomes of amentoflavone-treated *C. elegans*. **(A)** Volcano plot in control vs amentoflavone-treatment. Each point represents a single transcript. Plotted along the x-axis is the log2 (FC) of each transcript (transcript expression with or without amentoflavone-treated worms). The y-axis represents the negative logarithm of the corresponding *p*-value of that transcript. Up- and downregulated transcripts with p < 0.05 and log2FCI >1 are shown in red and green, respectively, whereas transcripts showing no differential expression are shown in grey; **(B)** Heatmap of significantly changed expression amounts of transcripts in control vs amentoflavone-treatment; **(C)** Amentoflavone significant KEGG pathways identified by enrichment analyses. **(D)** PPI network of top 5 DETs in control vs amentoflavone-treated *C. elegans*; **(E)** The effect of amentoflavone on the pharyngeal pumping frequency in N2 wild-type *C. elegans*. **(F)** The lifespan analysis of DF-16 mutant worms with amentoflavone compared to Control by one-way ANOVA following log-rank test.

To further explore the mechanisms by which *Selaginella* polyphenols extend lifespan, we performed RNA sequencing to analyze differentially expressed transcripts (DETs) in *C. elegans* treated with amentoflavone. We identified 693 DETs (372 upregulated, 267 downregulated) in amentoflavone-treated worms (Supplementary Table S13). Volcano plots and heatmaps of these DETs (FPKM >1, p < 0.05) are presented in Figures 4A,B.

Subsequent Gene Ontology (GO) enrichment analysis categorized these DETs into 3,214 terms, encompassing 2,161 biological processes (BPs), 402 cellular components (CCs), and 651 molecular functions (MFs) (Supplementary Table S14). The top 10 enriched terms for each category are shown in Supplementary Figure S9. The DETs were predominantly associated with BPs including regulation of biological process, cellular process, and response to stimulus. Key CCs included supramolecular complex and cell junction, while primary MFs involved metal ion binding and protein binding (Supplementary Figure S9; Supplementary Table S15).

KEGG pathway analysis revealed significant enrichment (p < 0.05) of these DETs in 8 pathways, among which 5 signaling pathways—MAPK, FOXO, Phosphatidylinositol, mTOR, and ECM-receptor interaction—were relevant to our study objectives (Figure 4C; Supplementary Table S16,S17). Gene set enrichment analysis (GSEA) indicated upregulation of all these signaling pathways (Supplementary Table S18).

To identify key molecular events, we conducted protein-protein interaction (PPI) analysis on DETs related to aging and longevity. Five central hub genes were identified: daf-16, daf-18, daf-15, egl-8, and lin-45 (Figure 4D; Supplementary Table S10). DAF-16, a key transcription factor downstream of insulin/IGF-1 signaling, regulates lifespan (Murphy et al., 2003). DAF-15 deficiency-induced longevity requires intestinal DAF-16/FOXO activity (Zang et al., 2024), while DAF-18 promotes DAF-16 nuclear translocation by inhibiting PIP3 and the PI3K-Akt pathway and is linked to antioxidant activity (Berman and Kenyon, 2006). Furthermore, other hub genes (egl-8, lin-45) are functionally connected to the FOXO pathway: EGL-8 acts upstream of DAF-16 to regulate lifespan in a DAF-16-dependent manner (Mack et al., 2022); LIN-45 (ERK signaling) influences DAF-2/DAF-16 insulin-like signaling via SKN-1 (Okuyama et al., 2010). These findings collectively suggest that amentoflavone extends lifespan by activating the DAF-16/FOXO transcription factor and upregulating the FOXO signaling pathway (Figures 4A–D).

### 3.7 Amentoflavone extends lifespan via DAF-16/FOXO-dependent antioxidant gene regulation in *C. elegans*

To exclude potential confounding effects of dietary restriction on the IIS signaling pathway in *C. elegans*, we measured the pharyngeal pumping rate of wild-type worms following amentoflavone treatment. As shown in Figure 4E, a slight increase in pharyngeal pumping frequency was observed in amentoflavone-treated worms compared to the control group, indicating that amentoflavone does not reduce feeding behavior. Moreover, we also assessed the healthspan of *C. elegans* following amentoflavone treatment, specifically focusing on body bending ability and reproductive capacity. However, the results revealed no significant differences in either body bending or fertility between the amentoflavone-treated group and the blank control group (Supplementary Figure S8).

**FIGURE 5:**
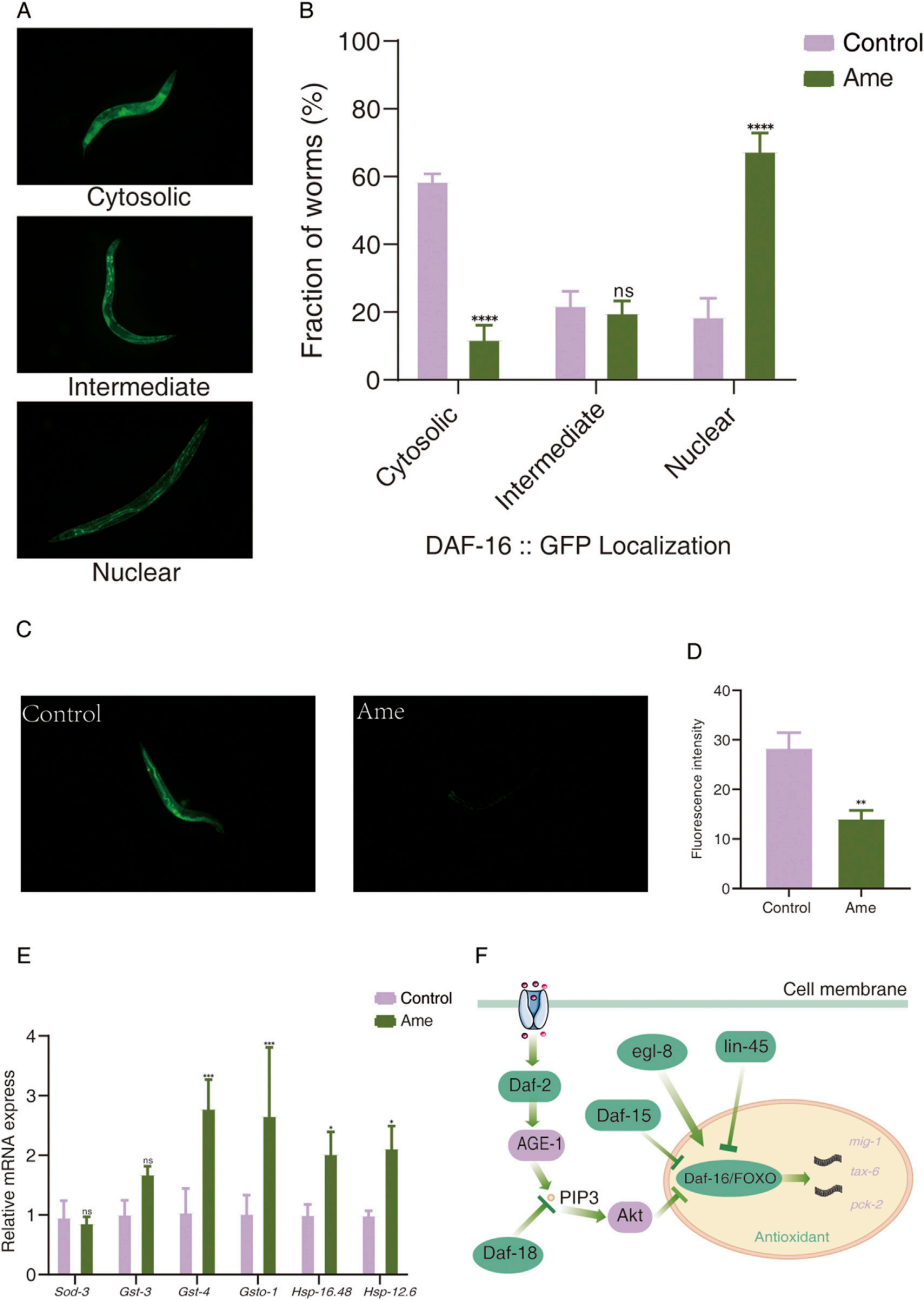
Exploration of the antioxidant mechanism of amentoflavone from *Selaginella* in delaying aging in *C. elegans*. **(A)** Representative fluorescence images showing three typical subcellular distributions of DAF-16 in transgenic strain TJ356: cytosolic localization, intermediate localization, and nuclear localization. **(B)** Effect of amentoflavone treatment on the subcellular localization of DAF-16 in TJ356 worms. *P* < 0.05, *P* < 0.01, *P* < 0.001 compared with the control group. **(C)** Fluorescence microscopy images of reactive oxygen species (ROS) accumulation. **(D)** Quantitative analysis of ROS fluorescence intensity. **(E)** mRNA expression levels of antioxidant-related genes in *C. elegans* after treatment with amentoflavone. **(F)** Proposed pathways involved in longevity regulation mediated by DAF-16/FOXO in amentoflavone-treated *C. elegans*. Ame, amentoflavone.

To further validate the pivotal role of DAF-16, we treated daf-16 knockout C. elegans mutants with amentoflavone (50μg mL-1). No significant alterations in survival curves were observed compared to wild-type controls (Figure 4F), confirming that DAF-16 is essential for the anti-aging effects of this compound. Since nuclear translocation is necessary for DAF-16 function, we used the DAF-16GFP (TJ356) strain to examine whether amentoflavone influences DAF-16 subcellular localization. As illustrated in Figures 5A,B, amentoflavone treatment significantly enhanced nuclear accumulation of DAF-16 by 48.85% compared to the control, supporting the involvement of this transcription factor in lifespan extension.

We next assessed intracellular ROS levels in *C. elegans*, as reduced ROS is indicative of enhanced antioxidant capacity. Amentoflavone administration resulted in a 50.22% decrease in ROS content compared to controls (Figures 5C,D).

Given the well-established association between FOXO signaling and antioxidant defense (Kim et al., 2014), we investigated DAF-16-interacting genes within protein–protein interaction (PPI) networks (Supplementary Figure S10). Our analysis revealed that DAF-16 interacts with three antioxidant-related genes: *mig-1, tax-6*, and *pck-2* (Supplementary Table S19). Expression analysis indicated that nearly all of these genes (with the exception of mig-1) were significantly upregulated (p < 0.05) following amentoflavone treatment.

To further assess the impact on antioxidant gene expression downstream of FOXO, we performed RT-qPCR on key genes, including *Sod-3, Gst-3, Gst-4, Gsto-1, Hsp-16.48,* and *Hsp-12.6* (Figure 5E). While *Sod-3* expression was only mildly increased, members of the *Hsp* and *Gst* families showed significant upregulation compared to the control group. These results suggest that amentoflavone enhances antioxidant defenses primarily through the induction of these gene families.

In conclusion, our findings indicate that amentoflavone extends lifespan and improves antioxidant capacity in *C. elegans* mainly by activating DAF-16/FOXO and upregulating its downstream antioxidant pathway. DAF-16/FOXO-mediated gene regulation appears crucial for the observed longevity phenotype (Figure 5F).

## 4 Discussion

Our study provides comprehensive evidence that *Selaginella* extracts, particularly those rich in the biflavonoid amentoflavone, significantly extend lifespan and enhance stress resilience in *C. elegans*. By integrating phytochemical analysis, transcriptomics, network pharmacology, and functional genetics, we demonstrate that amentoflavone is a key bioactive component responsible for these effects, primarily through the activation of the DAF-16/FOXO transcription factor and subsequent enhancement of antioxidant defense mechanisms.

The substantial variability in amentoflavone content across different *Selaginella* species and geographic origins—and its strong correlation with longevity promotion—highlights this compound as a critical mediator of the observed anti-aging effects. The high-amentoflavone extract S4 extended lifespan under both standard and UV-stress conditions, whereas the low-amentoflavone S16 exhibited reduced efficacy and signs of potential toxicity at higher concentrations. These findings underscore the importance of phytochemical standardization in herbal medicine research⁠.

Notably, purified amentoflavone alone extended median lifespan by 63.81%—significantly surpassing the effect of the crude extract and exceeding the efficacy of several well-known longevity compounds such as resveratrol (28.6%) and ginsenosides (34.1%)⁠ (Li et al., 2023), as well as the average lifespan extension reported for most traditional Chinese medicine ingredients (15.3%–31.3%)⁠ (Wang et al., 2021). This remarkable effect underscores the strong potential of amentoflavone for commercial development in nutraceuticals and cosmeceuticals⁠ (Wang et al., 2024). Moreover, amentoflavone conferred greater resistance to thermal and oxidative stress than to UV stress, suggesting that its mechanisms are particularly associated with mitigating oxidative damage and proteotoxic stress.

Transcriptomic and network pharmacology analyses revealed that both S4 extract and amentoflavone modulate evolutionarily conserved longevity pathways, including MAPK, mTOR, and FOXO signaling. The convergence of both treatments on FOXO pathway activation—supported by enrichment analyses and hub gene identification—emphasizes the importance of insulin/IGF-1 signaling (IIS) modulation in *Selaginella*-induced longevity. Specifically, amentoflavone promoted nuclear translocation of DAF-16 and upregulated the expression of antioxidant genes such as *gst-4*, *sod-3*, and heat shock protein family members. The potent antioxidant activity of amentoflavone and other *Selaginella* polyphenols is consistent with previous reports⁠ (Křížkovská et al., 2020; Kumar et al., 2021; Bailly, 2021; Muema et al., 2022). It is also noteworthy that amentoflavone did not reduce pharyngeal pumping rate, ruling out dietary restriction as a confounding factor. Instead, its effects are clearly linked to enhanced antioxidant capacity, as evidenced by significantly reduced ROS levels in treated worms. These findings align with existing literature on the role of DAF-16 in regulating oxidative stress response and longevity⁠ (Rodriguez-Colman et al., 2024; Cho and Park, 2024).

While our work clearly establishes amentoflavone as a key longevity-promoting compound in *Selaginella*, several questions remain. For instance, the broader ecological and genetic factors influencing amentoflavone accumulation in *Selaginella* species merit further investigation. Moreover, the precise molecular interactions through which amentoflavone activates DAF-16—whether through direct modulation of upstream regulators like DAF-2 or through alternative pathways—require deeper mechanistic inquiry. The potential synergy between amentoflavone and other phytochemicals in *Selaginella* also warrants additional study (Wang et al., 2024).

Beyond *C. elegans*, the conservation of the IIS/FOXO pathway across metazoans suggests that amentoflavone may offer therapeutic potential for aging-related disorders in mammals. Future studies should validate these findings in murine models and explore possible applications in delaying age-related decline or treating oxidative-stress-related pathologies (Chen et al., 2021).

In conclusion, our results position *Selaginella* and its constituent amentoflavone as promising candidates for developing natural interventions aimed at promoting healthy aging. By elucidating the genetic and pharmacological mechanisms underlying their effects, this work bridges traditional herbal medicine and contemporary molecular gerontology, offering a robust foundation for future research and application.

## Data Availability

The transcriptome data presented in the study are deposited in the The Genome Sequence Archive (GSA), accession number CRA031806. The raw contributions presented in the study are publicly available. This data can be found here: https://figshare.com/s/98776ec4c63aaf336209.
