# Supplementary material for: Selaginella extracts extend lifespan and mitigate oxidative stress in Caenorhabditis elegans
Source: Front Pharmacol. 2025 Nov 25;16:1658991. doi: 10.3389/fphar.2025.1658991 (PMC12687336; doi:10.3389/fphar.2025.1658991)
Supplement: Supplementary file 1 [file Supplementaryfile1.pdf]

Supporting information

This Supporting information file includes:

1. Supporting Methods

2. Supporting Results

3. Supplementary Figures 1-10

4. Supplementary Tables S1, S3, S10, S11, S12, S13, S16, S18, S19, S20

5. Additional Data Supporting Tables (separate file)

1 Supporting Methods

Network pharmacology analysis

To systematically investigate the multi-component and multi-target mechanisms underlying the anti-aging effects of *Selaginella*, a network pharmacology approach was employed.

Compound collection and target prediction

Chemical constituents of *Selaginella* were retrieved from the INPUT (http://cbcb.edutcm.edu.cn/INPUT/) and HERB (http://herb.ac.cn/) databases. Potential protein targets of these compounds were predicted using SwissTargetPrediction (https://www.swisstargetprediction.ch/), with a probability threshold of ≥50% for target inclusion.

Aging-related targets

Aging-related genes were collected from GeneCards (https://www.genecards.org/), TTD (http://db.idrblab.net/ttd/), and DrugBank (https://go.drugbank.com/) using the keyword "aging." Targets with a relevance score ≥5 in GeneCards were retained. Redundant entries were removed to generate a non-redundant aging-related gene set.

Intersection analysis

Common targets between *Selaginella* compounds and aging-related genes were identified using Venny 2.1.0 (https://bioinfogp.cnb.csic.es/tools/venny/) and visualized via a Venn diagram.

Protein-Protein Interaction (PPI) network construction

PPI networks were built using the STRING database (https://string-db.org/) with "Homo sapiens" as the species. Interactions with a confidence score ≥0.4 were included. Isolated nodes were removed. The PPI network was visualized and analyzed using Cytoscape 3.10.1.

Network construction and key component identification

A "Disease-Component-Target" network was constructed to visualize interactions between aging, *Selaginella* components, and their targets. Key bioactive components were identified based on degree centrality calculated using CytoHubba.

Functional enrichment analysis

Gene Ontology (GO) and Kyoto Encyclopedia of Genes and Genomes (KEGG) pathway enrichment analyses were performed for the common targets using the DAVID database (https://david.ncifcrf.gov/). Results were visualized via the bioinformatics online platform (http://www.bioinformatics.com.cn/).

Molecular docking

The 3D structures of key components (amentoflavone and selaginellin) were obtained from PubChem (https://pubchem.ncbi.nlm.nih.gov/). Crystal structures of top target proteins (e.g., STAT3, ESR1, HSP90AA1, HIF1A, NFkB1, GSK3B, MTOR, PTGS2, SIRT1, TLR4) were downloaded from the PDB (https://www.rcsb.org/). Molecular docking was performed using AutoDock Vina (v1.1.2) to evaluate binding affinities (kcal/mol).

2 Supporting Results

Multi-component and multi-target mechanisms of *Selaginella* in aging regulation

Selaginella is rich in diverse secondary metabolites, including alkaloids, flavonoids, terpenoids, and phenolic compounds. To identify the key anti-aging components, we integrated network pharmacology and molecular docking approaches.

A total of 38 chemical components were identified from *Selaginella*, targeting 393 potential proteins. Among these, 181 targets overlapped with aging-related genes (Fig. S4A). GO enrichment analysis revealed significant involvement in biological processes such as positive regulation of transcription by RNA polymerase II, cellular components including plasma membrane, and molecular functions such as protein binding (Fig. S5, Table S11). KEGG analysis highlighted the insulin resistance pathway as the most enriched (Fig. S4B).

The "Disease-Component-Target" network comprised 211 nodes and 1,923 edges (Fig. S6). Ten key components were identified based on degree centrality: andromedotoxin, asebotoxin, β-caryophyllene, isocembrol, hinokiflavone, isocryptomerin, amentoflavone, selaginellin, hinokinin, and tremetone (Table S12). These include phytotoxins, terpenes, flavonoids, and phenylpropanoids, suggesting a multi-component mechanism.

PPI network analysis of the 181 common targets identified 1,603 interactions (Fig. S7). The top 10 hub targets were STAT3, ESR1, HSP90AA1, HIF1A, NFkB1, GSK3B, MTOR, PTGS2, SIRT1, and TLR4 (Fig. S4C, Table S13), all of which are well-documented regulators of aging.

Molecular docking confirmed strong binding affinities (≤ –5.4 kcal/mol) between the key components and these targets (Fig. S4D, S4E), supporting the potential of *Selaginella* to modulate aging through a multi-target, multi-component network.

These results provide a systematic foundation for understanding the anti-aging properties of *Selaginella* and highlight amentoflavone as a key bioactive compound worthy of further investigation.

3 Supporting Figures

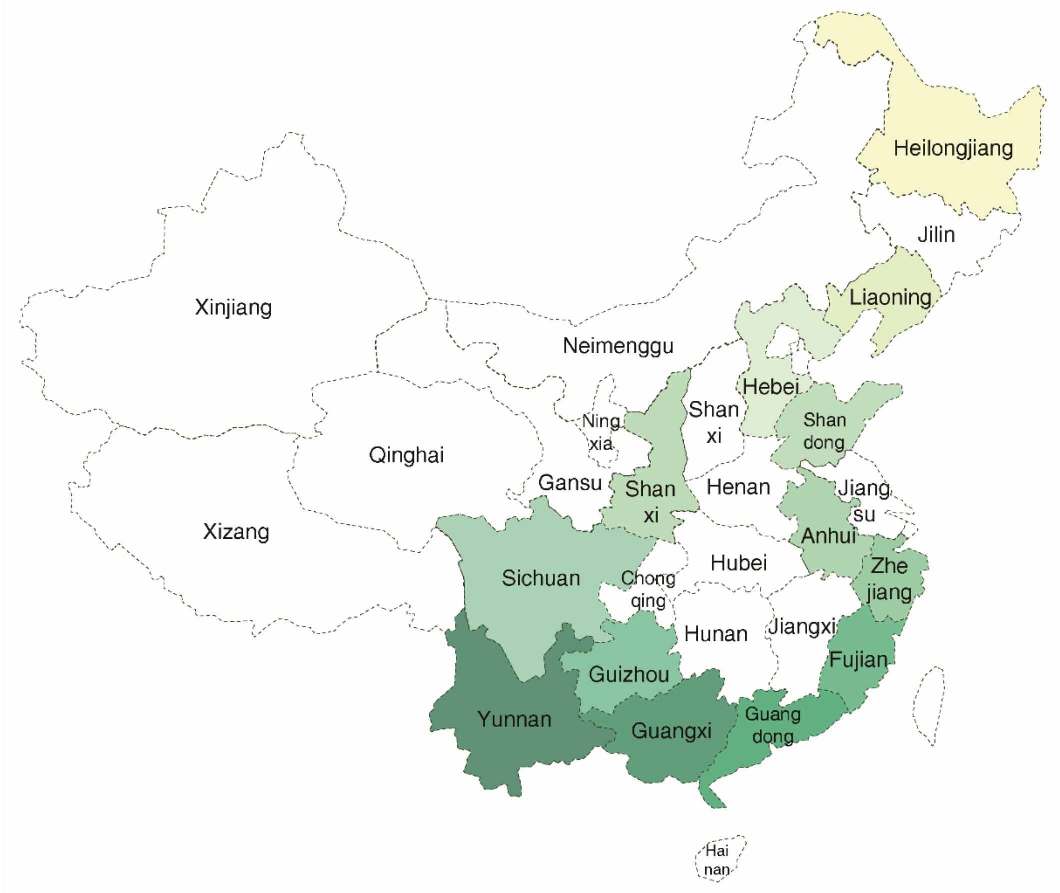

Figure S1. Geographic distribution of *Selaginella* sample collections across China.

Fill colors represent provinces from which *Selaginella* samples were collected; white indicates no samples were obtained from that region.

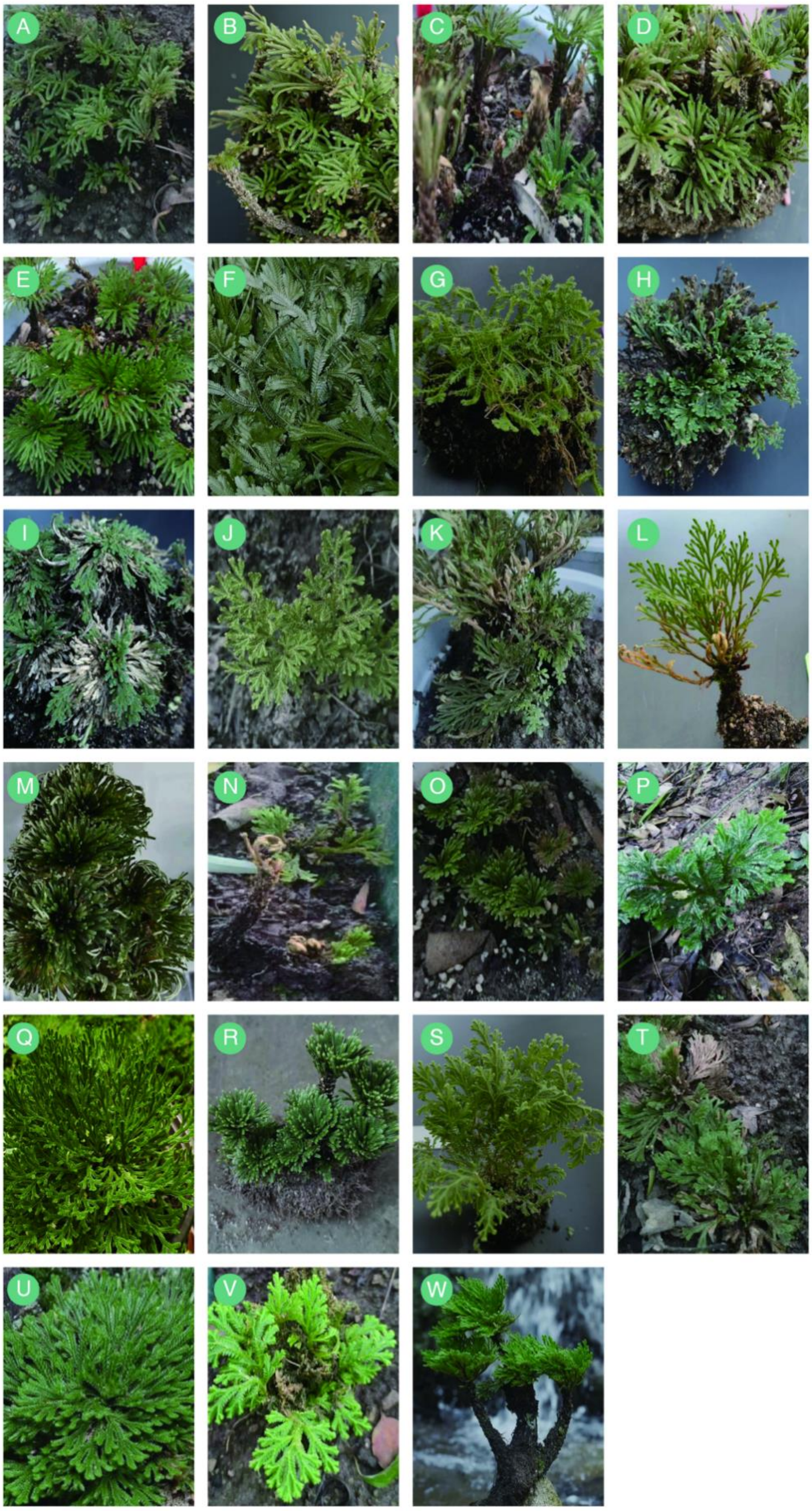

Figure S2. Morphological characteristics of the 23 *Selaginella* samples.

Images **A–W** correspond to samples S1–S23, respectively, as listed in Table S1.

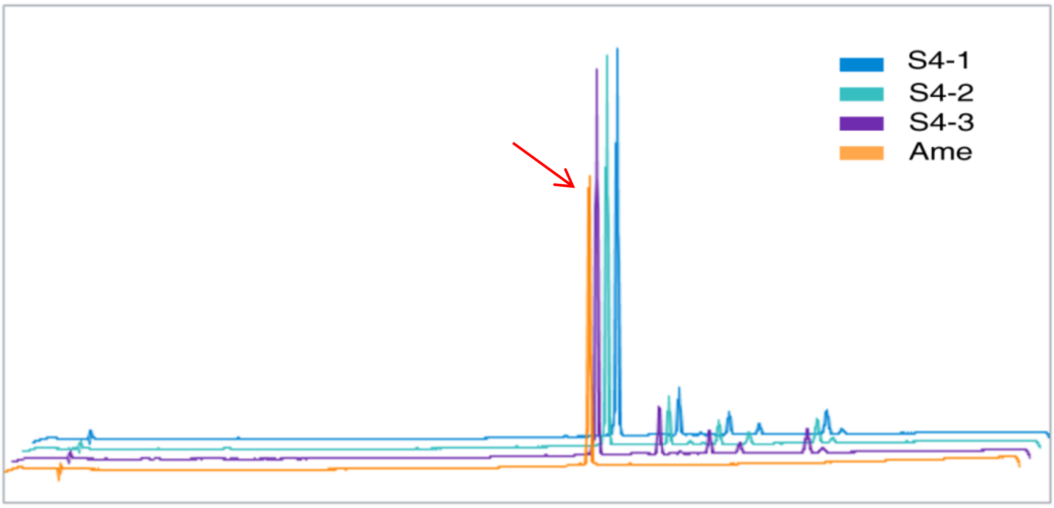

Figure S3. HPLC chromatogram of the S4 methanol extract.

The peak corresponding to amentoflavone is indicated by the arrow. Amentoflavone was identified by comparing its retention time and UV spectrum with those of an authentic standard.

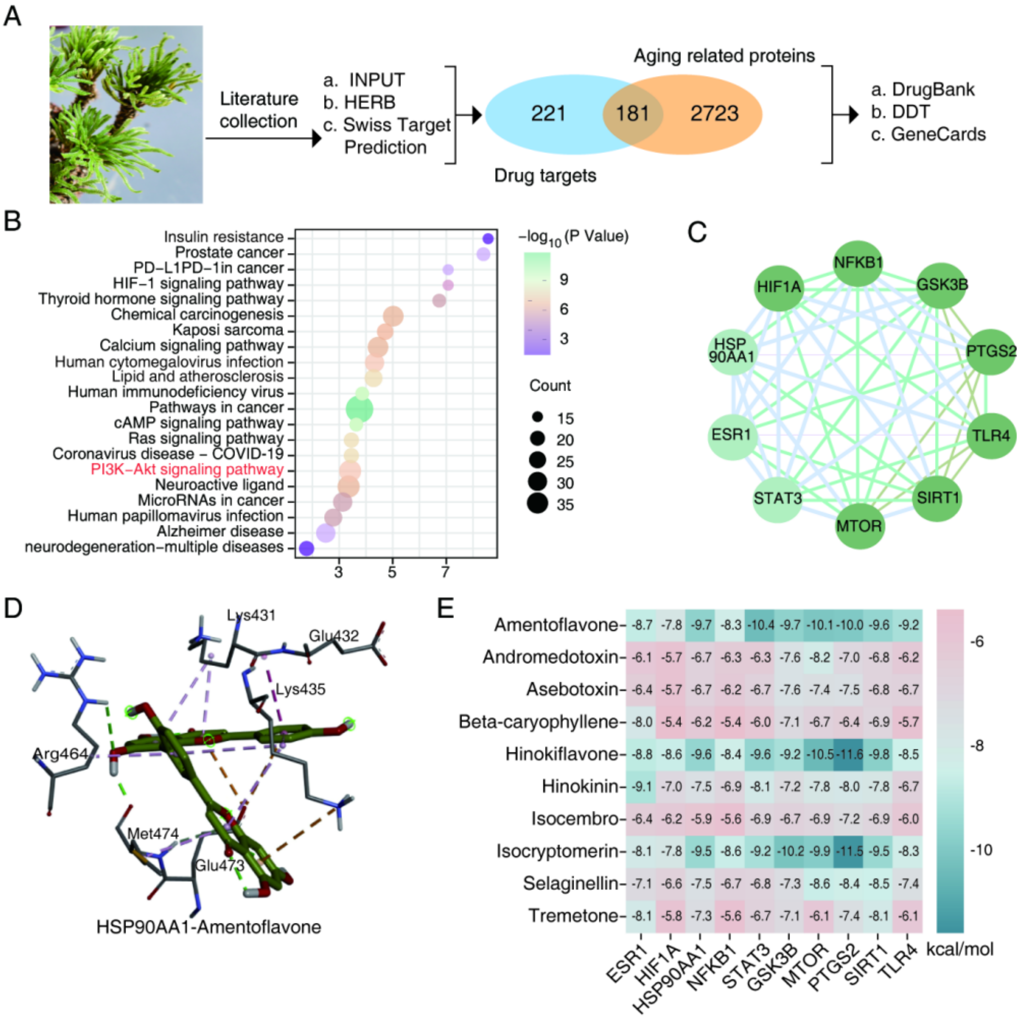

Figure S4. Network pharmacology analysis of *Selaginella* for aging-related targets.

(A) Workflow for identifying *Selaginella* components and aging-related targets, and Venn diagram showing their intersection.

(B) KEGG pathway enrichment analysis of the 181 intersecting targets.

(C) Protein-protein interaction (PPI) network of the top 10 aging-related targets.

(D) Binding affinities estimated by molecular docking (kcal·mol^{-1}) of key *Selaginella* components with the top 10 targets.

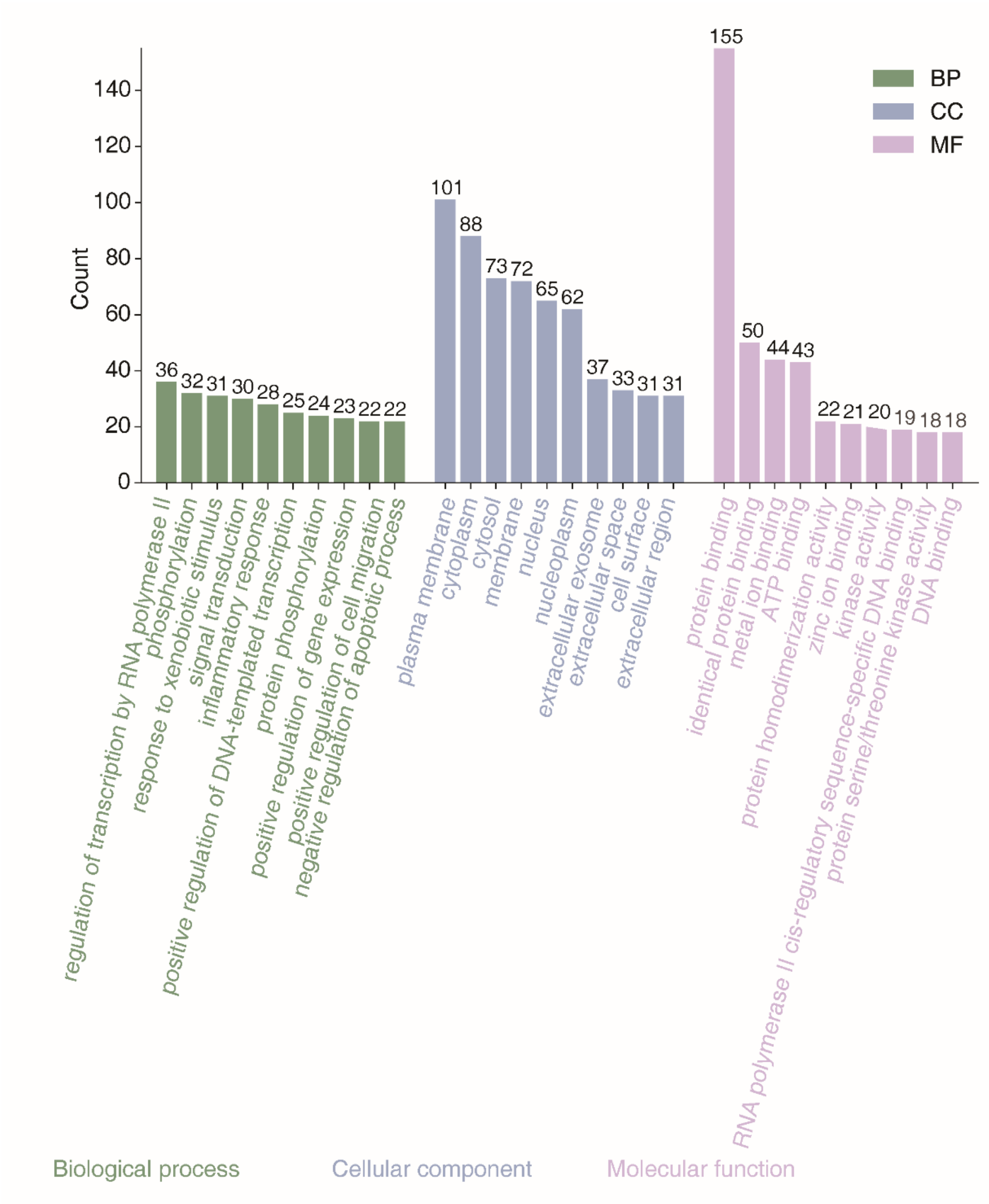

Figure S5. Gene Ontology (GO) enrichment analysis of targets associated with the top 10 anti-aging components of *Selaginella*.

Top 10 enriched terms in biological process, cellular component, and molecular function categories are shown.

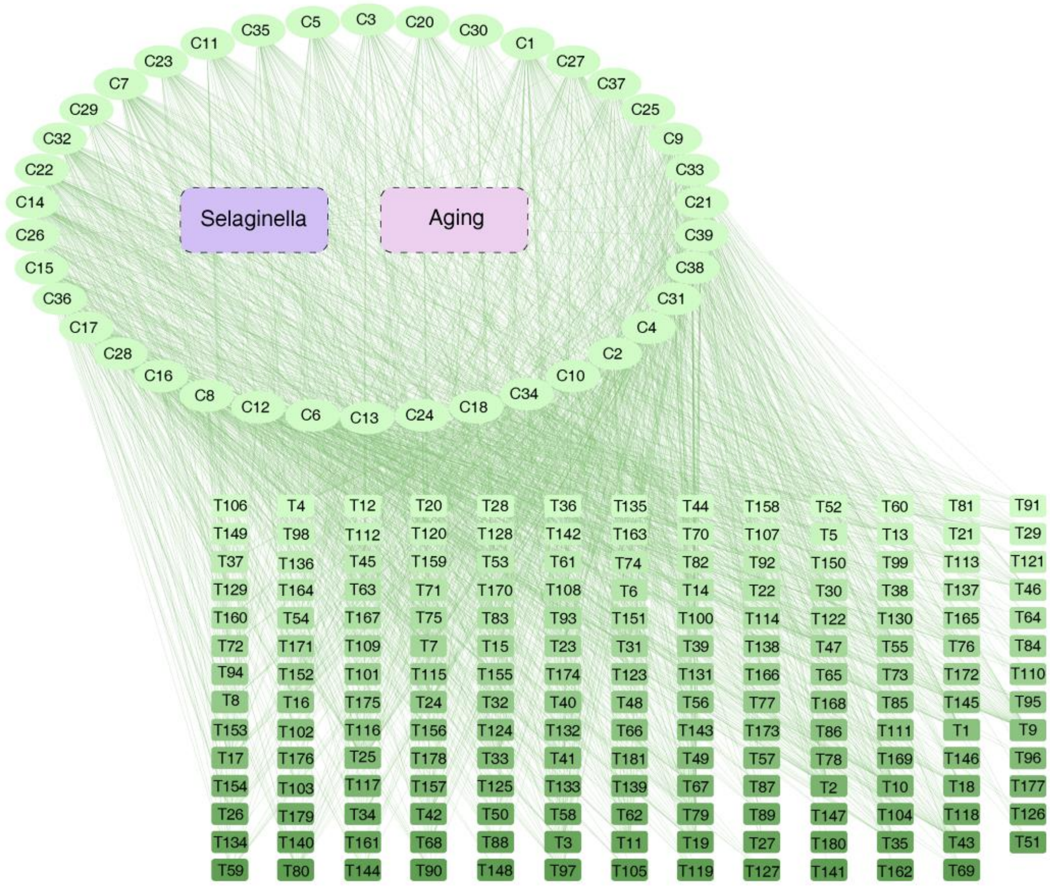

Figure S6. "Disease-Drug-Component-Target" network for *Selaginella* in aging.

Nodes represent diseases, drugs, components, and targets; edges indicate interactions. Key components are highlighted based on degree centrality.

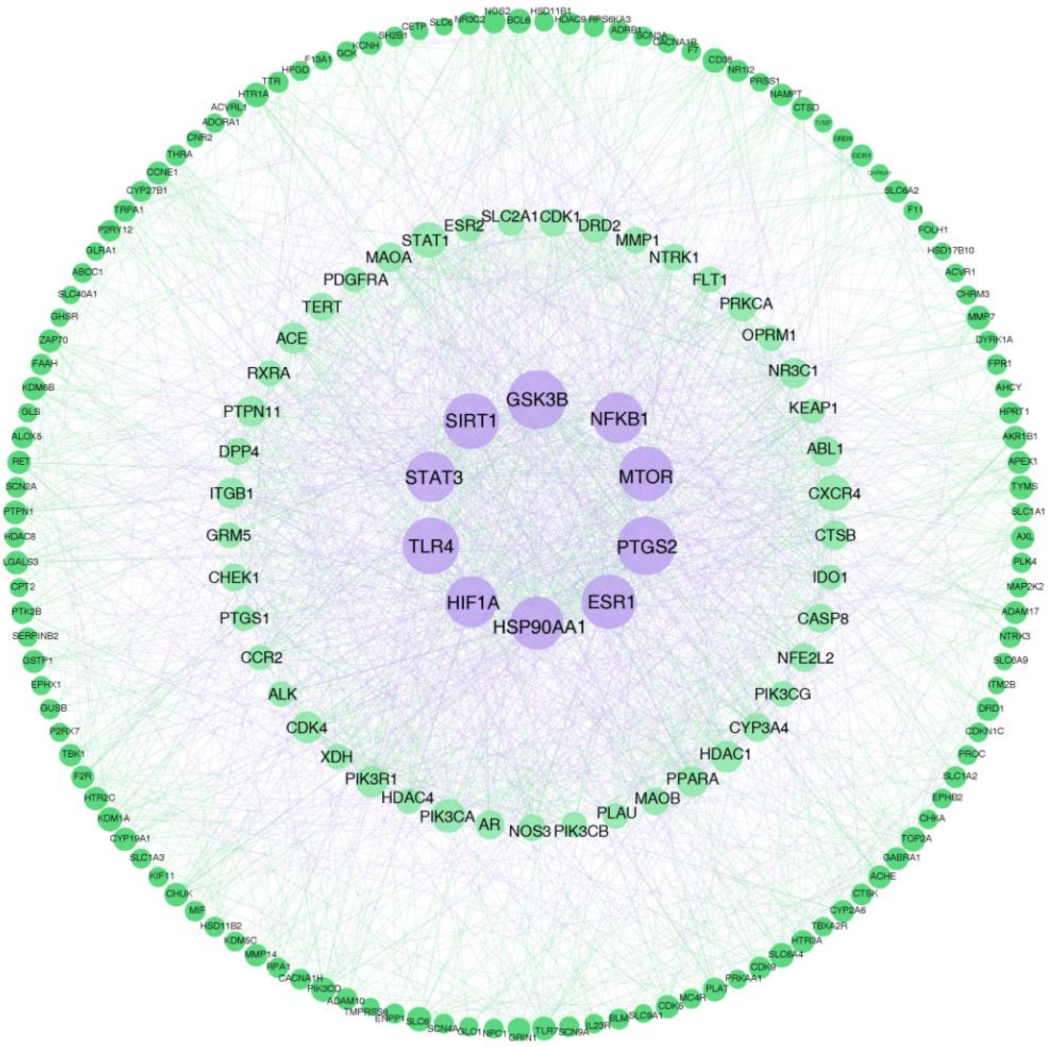

Figure S7. Protein-protein interaction (PPI) network of the 181 intersecting targets between *Selaginella* components and aging-related genes.

Nodes represent proteins; edges represent interactions with a confidence score ≥ 0.4.

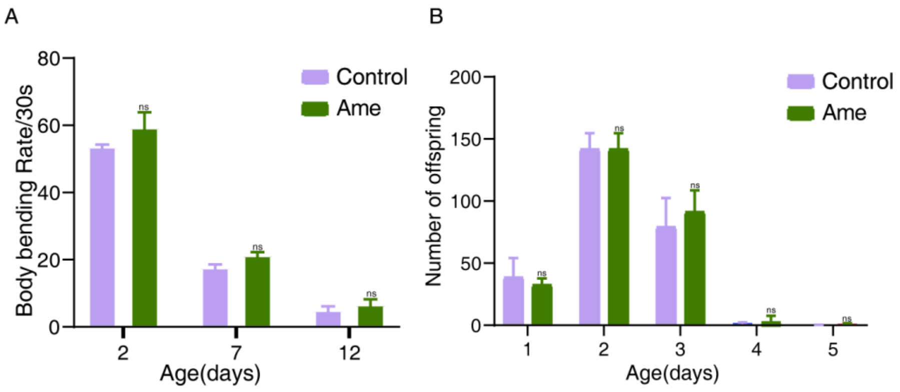

Figure S8. Effect of amentoflavone on age-related phenotypes in *C. elegans*.

(**A**) **Body bending rate**: The effect of amentoflavone (50 μg mL^{-1}) on the motility of N2 wild-type *C. elegans* was assessed after 2, 7, and 12 days of treatment. Motility was quantified by counting the number of body bends within a 30-second interval.

(**B**) **Fecundity assay**: The influence of amentoflavone (50 μg mL^{-1}) on reproductive capacity was evaluated. Data are expressed as mean ± SD.

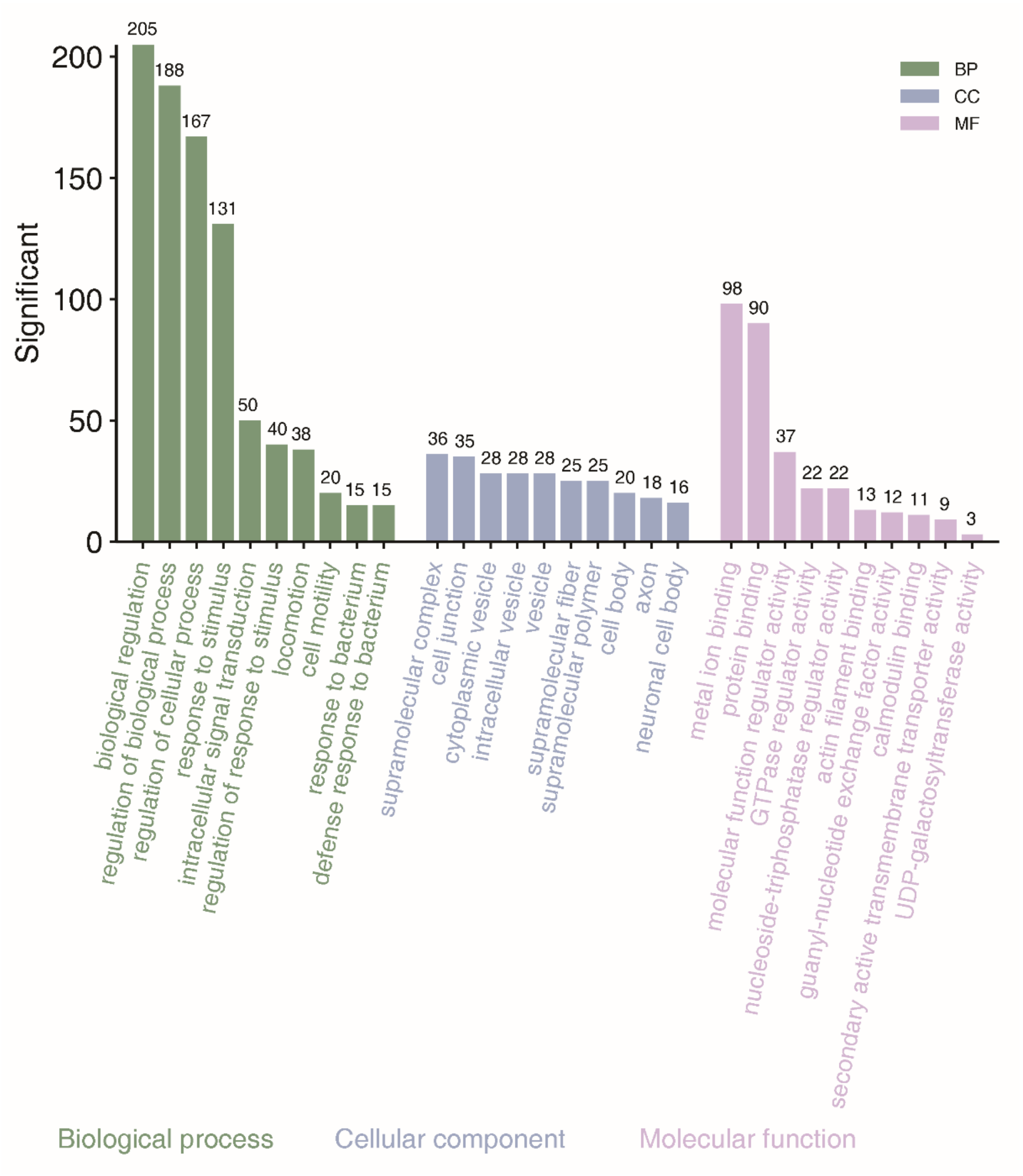

Figure S9. Top 10 enriched Gene Ontology (GO) terms for differentially expressed transcripts (DETs) in *C. elegans* treated with amentoflavone.

Terms are grouped into biological process, cellular component, and molecular function categories.

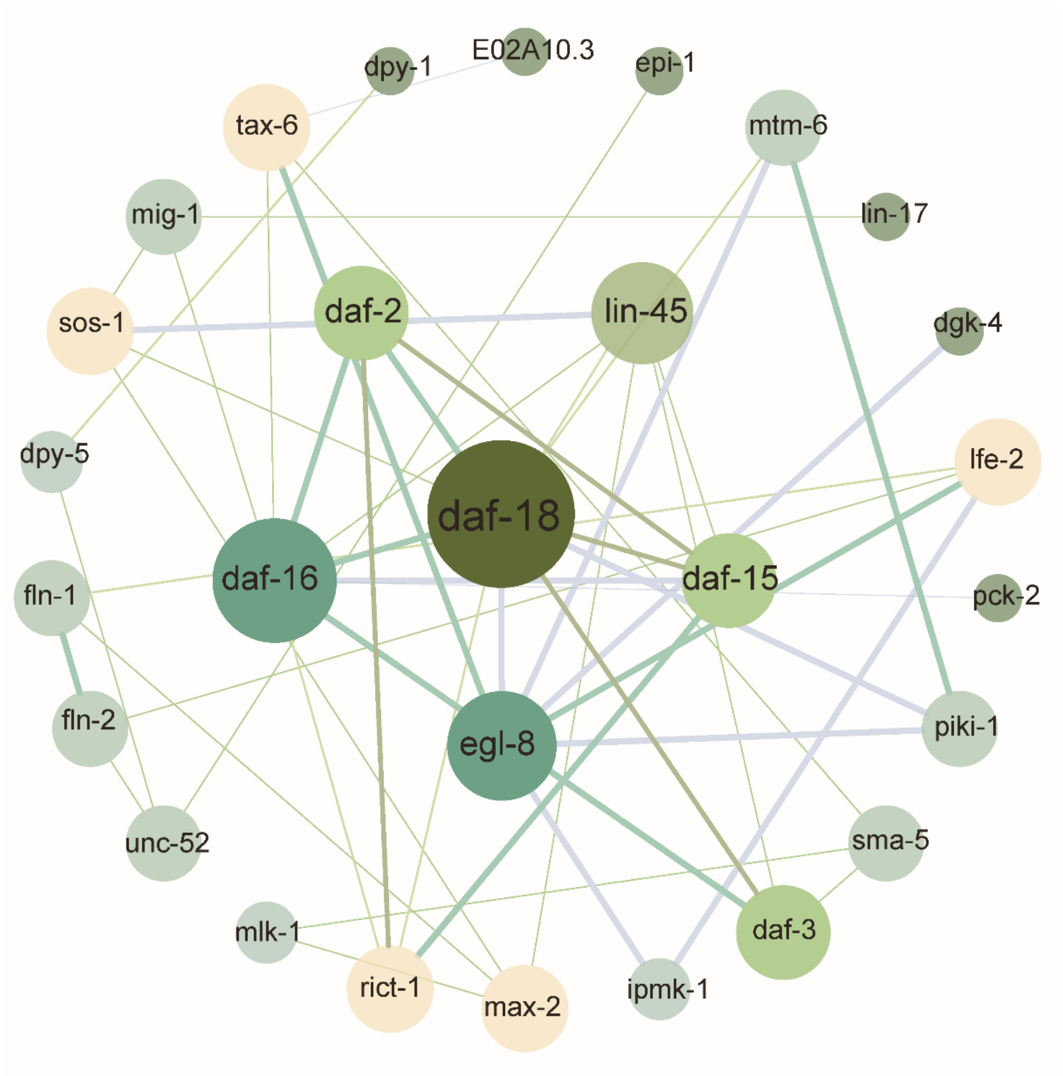

Figure S10. Protein-protein interaction (PPI) network of differentially expressed transcripts (DETs) in *C. elegans* treated with amentoflavone.

Hub genes related to aging and antioxidant response are highlighted.

4 Supporting Tables

Table S1. Collection details and geographical origins of the 23 *Selaginella* samples used in this study.

NO.	Species	Provinces	Locations
S1	*S. tamariscina* (P.Beauv.) Spring	Guangdong	Shaoguannanxiong
S2			Shaoguanrenhua
S3			Shaoguan
S4			Shaoguanshixing
S5			Chaoshan
S6	*S. moellendorffii* Hieron.	Guangxi	Yulin
S7	*S. davidii* Franch.	Guizhou	Anshunziyun
S8	*S. pulvinata* (Hook. & Grev.) Maxim.	Yunnan	Lijiang
S9		Sichuan	Leshan
S10	*S. martensii* Spring	Zhejiang	Lishui
S11	*S. tamariscina* (P.Beauv.) Spring		Taizhou
S12			Hangzhou
S13			Taizhouwenling
S14			Jinhua
S15	*S. tamariscina* (P.Beauv.) Spring	Fujian	Xiamen
S16	S. doederleinii Hieron.		Nanpingpu
S17	*S. pulvinata* (Hook. & Grev.) Maxim.	Anhui	Bozhou
S18	*S. tamariscina* (P.Beauv.) Spring	Jiangsu	Suqian
S19		Shandong	Weifang
S20	*S. pulvinata* (Hook. & Grev.) Maxim.	Liaoning	Dandong
S21		Heilongjiang	Yichun
S22	*S. martensii* Spring	Hebei	Zhangjiakou
S23	*S. tamariscina* (P.Beauv.) Spring	Jiangsu	Shuyang

Table S3. Amentoflavone content classification of 23 *Selaginella* samples into high, medium, and low groups.

Group	**No.**	Amentoflavone content (μg g^{-1})
High-amentoflavone group	S11	33.91
	S4	33.07
	S12	25.40
	S15	25.03
	S2	24.62
	S23	25.28
	S18	23.92
	S1	23.88
	S5	23.49
Medium-amentoflavone group	S10	19.51
	S14	19.19
	S3	19.12
	S13	17.56
	S8	12.98
	S21	11.82
	S7	11.15
Low-amentoflavone group	S9	10.39
	S20	8.78
	S17	8.73
	S19	6.71
	S22	6.05
	S6	6.03
	S16	4.24

Table S10. Chemical components of *Selaginella* retrieved from public databases with predicted anti-aging targets.

No.	Name	Degree	Category
C7	Andromedotoxin	76	Alkaloids
C22	Beta-caryophyllene	75	Sesquiterpenes
C32	Asebotoxin	75	Alkaloids
C14	Isocembrol	70	Sesquiterpenes
C1	Hinokiflavone	62	Flavonoids
C33	Isocryptomerin	59	Terpenes
C2	Amentoflavone	58	Flavonoids
C9	Hinokinin	55	Alkaloids
C38	Selaginellin	55	Phenolics
C6	Germanicol	53	Coumarins
C8	Fastigilin B	53	Lkaloids
C23	Tremetone	53	Flavonoids
C11	Azaleatin	52	Alkaloids
C17	Apigenin	52	Flavonoids
C21	Alpha-Selinene	52	Terpenes
C4	Quercitrin	51	Flavonoids
C15	Kaempferol	51	Flavonoids
C5	Avicularin	50	Flavonoids
C18	4-Hydroxy mephenytion	50	Coumarins
C27	Scopoletin	50	Coumarins
C35	(S)-4-Hydroxy Mephenytoin	50	Coumarins
C3	Isohyperoside	49	Flavonoids
C12	Taxifolin	48	Flavonoids
C19	Dihydroresveratrol	45	Phenolics
C28	Caffeic acid	45	Phenolics
C29	Vanillic acid	45	Phenolics
C36	3,4-Dihydroxycinnamic Acid	45	Phenolics
C37	Acide Vanillique	45	Phenolics
C13	Quercetin	44	Flavonoids
C20	Germacrone	44	Sesquiterpene
C26	FER	44	Protein
C16	Isoimperatorin	43	Coumarins
C25	Syringic acid	41	Phenolics
C24	Cedar acid	40	Phenolics
C34	Trans-Ferulic Acid	40	Phenolics
C10	Gossypetin	39	Flavonoids
C31	Hydroquinone	39	Phenolics

C30	Benzylacetone	28	Aromatic compounds

Table S11. Gene Ontology (GO) enrichment analysis of top 10 anti-aging components from *Selaginella*.

Term	Subgroup	Count
Positive regulation of transcription by RNA polymerase II	Biological process	36
Phosphorylation		32
Response to xenobiotic stimulus		31
Signal transduction		30
Inflammatory response		28
Positive regulation of DNA-templated transcription		25
Protein phosphorylation		24
Positive regulation of gene expression		23
Positive regulation of cell migration		22
Negative regulation of apoptotic process		22
Plasma membrane	Cellular component	101
Cytoplasm		88
Cytosol		73
Membrane		72
Nucleus		65
Nucleoplasm		62
Extracellular exosome		37
Extracellular space		33
Cell surface		31
Extracellular region		31
Protein binding	Molecular function	155
Identical protein binding		50
Metal ion binding		44
ATP binding		43
Protein homodimerization activity		22
Zinc ion binding		21
Kinase activity		20
RNA polymerase II cis-regulatory region sequence-specific DNA binding		19
Protein serine/threonine kinase activity		18
DNA binding		18

Table S12. Top 10 key components of *Selaginella* identified by network pharmacology for anti-aging activity.

No.	Name	Degree
C7	Andromedotoxin	76
C32	Asebotoxin	75
C22	Beta-caryophyllene	75
C14	Isocembrol	70
C1	Hinokiflavone	62
C33	Isocryptomerin	59
C2	Amentoflavone	58
C38	Selaginellin	55
C9	Hinokinin	55
C23	Tremetone	53

Table S13. Top 10 potential anti-aging targets of *Selaginella* based on protein-protein interaction (PPI) network analysis.

No.	Name	Degree
T89	STAT3	87
T133	ESR1	74
T21	HSP90AA1	71
T76	HIF1A	66
T74	NFkB1	65
T143	GSK3B	63
T170	PTGS2	62
T82	TLR4	57
T111	SIRT1	55
T62	MTOR	52

Table S16. Top enriched Gene Ontology (GO) terms for differentially expressed transcripts (DETs) in *C. elegans* treated with amentoflavone.

GO term	Subgroup	Significant
biological regulation	Biological process	205
regulation of biological process		188
regulation of cellular process		167
response to stimulus		131
intracellular signal transduction		50
regulation of response to stimulus		40
locomotion		38
cell motility		20
response to bacterium		15
defense response to bacterium		15
supramolecular complex	Cellular component	36
cell junction		35
cytoplasmic vesicle		28
intracellular vesicle		28
vesicle		28
supramolecular fiber		25
supramolecular polymer		25
cell body		20
axon		18
neuronal cell body		16
metal ion binding	Molecular function	98
protein binding		90
molecular function regulator activity		37
GTPase regulator activity		22
nucleoside-triphosphatase regulator activity		22
actin filament binding		13
guanyl-nucleotide exchange factor activity		12
secondary active transmembrane transporter activity		11
calmodulin binding		9
UDP-galactosyltransferase activity		3

Table S18. KEGG pathway enrichment analysis of aging-related differentially expressed transcripts (DETs) in amentoflavone-treated *C. elegans*

ID	Description	pvalue	Count	Rich_factor
cel04010	MAPK signaling pathway	0.0074	19	0.0418
cel04068	FoxO signaling pathway	0.0191	13	0.0433
cel04070	Phosphatidylinositol signaling system	0.0429	9	0.0441
cel04150	mTOR signaling pathway	0.0252	12	0.0430
cel04512	ECM-receptor interaction	0.0376	8	0.0476

Table S19. Gene Set Enrichment Analysis (GSEA) of signaling pathways in amentoflavone-treated *C. elegans*

**ID**	**Description**	**P value**	**NES**
cel04010	MAPK signaling pathway	0.0074	1.6138
cel04068	FoxO signaling pathway	0.0191	1.7469
cel04070	Phosphatidylinositol signaling system	0.0429	1.3622
cel04150	mTOR signaling pathway	0.0252	1.4563
cel04512	ECM-receptor interaction	0.0376	1.3252

NES, greater than 0 is up and less than 0 is down

Table S20. Expression changes of antioxidant-related genes in *C. elegans* following amentoflavone treatment.

Gene ID	Name	LogFC	P-Value	Type	Description
Y34D9B.1	mig-1	1.1421	0.0308	Down	Cytochrome P450
R11A5.4	pck-2	2.2137	5.1E-06	Up	phosphoenolpyruvate carboxykinase (GTP)
C02F4.2	tax-6	1.1834	0.0186	Up	Serine/threonine-protein phosphatase 2B catalytic subunit
